# Transcriptional Changes in *Schistosoma mansoni* during Early Schistosomula Development and in the Presence of Erythrocytes

**DOI:** 10.1371/journal.pntd.0000600

**Published:** 2010-02-09

**Authors:** Geoffrey N. Gobert, Mai H. Tran, Luke Moertel, Jason Mulvenna, Malcolm K. Jones, Donald P. McManus, Alex Loukas

**Affiliations:** 1 Division of Infectious Diseases, Queensland Institute of Medical Research, Herston, Queensland, Australia; 2 School of Veterinary Sciences, The University of Queensland, Brisbane, Queensland, Australia; University of Pittsburgh, United States of America

## Abstract

**Background:**

Schistosomes cause more mortality and morbidity than any other human helminth, but control primarily relies on a single drug that kills adult worms. The newly transformed schistosomulum stage is susceptible to the immune response and is a target for vaccine development and rational drug design.

**Methodology/Principal Findings:**

To identify genes which are up-regulated during the maturation of *Schistosoma mansoni* schistosomula *in vitro*, we cultured newly transformed parasites for 3 h or 5 days with and without erythrocytes and compared their transcriptional profiles using cDNA microarrays. The most apparent changes were in the up-regulation of genes between 3 h and 5 day schistosomula involved in blood feeding, tegument and cytoskeletal development, cell adhesion, and stress responses. The most highly up-regulated genes included a tegument tetraspanin *Sm-tsp-3* (1,600-fold up-regulation), a protein kinase, a novel serine protease and serine protease inhibitor, and intestinal proteases belonging to distinct mechanistic classes. The inclusion of erythrocytes in the culture medium resulted in a general but less pronounced increase in transcriptional activity, with the highest up-regulation of genes involved in iron metabolism, proteolysis, and transport of fatty acids and sugars.

**Conclusions:**

We have identified the genes that are up-regulated during the first 5 days of schistosomula development *in vitro*. Using a combination of gene silencing techniques and murine protection studies, some of these highly up-regulated transcripts can be targeted for future development of new vaccines and drugs.

## Author Summary

Schistosome blood flukes cause more mortality and morbidity than any other human worm infection, but current control methods primarily rely on a single drug. There is a desperate need for new approaches to control this parasite, including vaccines. People become infected when the free-swimming larva, the cercaria, enters through the skin and becomes the schistosomulum. Schistosomula are susceptible to immune responses during their first few days in the host before they become adult parasites. We characterised the genes that these newly transformed parasites switch on when they enter the host to identify molecules that are critical for survival in the human host. Some of these highly up-regulated genes can be targeted for future development of new vaccines and drugs.

## Introduction

The schistosome tegument, an unique double lipid bilayered syncitium that covers the external surface of the intra-mammalian developmental stages, represents the point of interaction between the parasite and mammalian host tissues. This structure is pivotal for parasite survival within the host and is therefore a primary target of anthelmintic drugs [1] and vaccines [2],[3]. In similar fashion, the intestine, or gastrodermis of schistosomes is a source of secreted proteins and another point of interaction with host tissues (i.e. blood). The *Schistosoma mansoni* genome sequence has recently been reported [4] and the secreted proteome (secretome) has also been characterised with a major focus on the proteins present in the tegument and excretory/secretory (ES) products [4],[5],[6],[7],[8],[9]. While the schistosome gastrodermal proteome has not yet been explored, we recently described tissue-specific gene profiling for adult *S. japonicum* and characterised the transcriptome of gastrodermal cells using a combination of laser microdissection microscopy followed by cDNA microarray analysis [10]. Despite the progress made in characterising the mRNA and protein compositions of cells at the host-parasite interface, it is only now with the recent application of gene silencing technologies to the study of schistosomes, that we are understanding the functions of these proteins and how they enable schistosomes to exist as parasites [11].

In terms of vaccine development, the newly transformed schistosomulum is widely viewed as the most susceptible stage to antibody-mediated damage [2],[3],[12],[13],[14]. After cercariae transform into schistosomula, parasites undergo changes in their surface protein composition [15]. The schistosomula surface is dynamic, with some proteins appearing and others disappearing [16] as the parasites mature during their migration to the lungs. Once the parasites reach the lungs they are refractory to antibody-mediated damage [17] and cloak themselves in host blood group antigens [18] and other proteins involved in immune responses [19],[20].

Obtaining sufficient quantities of schistosomula directly from lung tissue for most research purposes is time consuming and involves working with mammalian hosts. As a result, many researchers mechanically transform cercariae and culture them in serum-containing medium [21]. This *in vitro* strategy also confers uniformity in parasite maturation, which is critical for short culturing periods and cannot be achieved *in vivo* due to the variation in the time required for individual parasites to penetrate host skin and enter the vasculature. Erythrocytes are not usually included in the culture medium yet the parasites are bathed in blood *in vivo*. Moreover, schistosomula acquire erythrocyte [22] and other cellular [18] proteins onto their teguments, and this is thought to aid in evasion of the immune response. Despite their vascular existence, the effect of erythrocytes on gene expression of *in vitro* cultured parasites has not been addressed until now.

We sought to identify the transcriptional changes in genes encoding surface and secreted proteins during the first 5 days of *in vitro* culture of schistosomula in the presence or absence of erythrocytes. Some of these surface exposed proteins are proving to be efficacious vaccines [23],[24], yet the expression profiles of only some of these genes have been explored, and have involved using arrays covering only ∼3,000 genes [25]. Here we show that the major transcriptional changes which occur during this 5 day time period involve a wide range of biological functions but prominent processes include tegument maturation, cellular development and organisation, gut function/nutrient acquisition and stress responses. The data provide a framework by which to select targets for vaccine and drug design based on genes that are critical for the development of *S. mansoni* larvae during their first few days in the mammalian host.

## Materials and Methods

### Culture of *S. mansoni* and total RNA isolation

The Puerto Rican strain of *S. mansoni* and *Biomphalaria glabrata* snails were provided by the National Institutes of Allergy and Infectious Diseases Schistosomiasis Resource Centre at the Biomedical Research Institute (Rockville, Maryland, USA). To obtain cercariae, *B. glabrata* snails infected with miracidia were exposed to incandescent light for 1 h and then washed twice in RPMI 1640, 1% antibiotic/antimycotic and 10 mM Hepes (Invitrogen). Cercariae were passed through a 22-gauge emulsifying needle 25 times to mechanically shear the cercarial tails from the bodies [26]. The resulting schistosomula were isolated from free tails by centrifugation through a 60% percoll gradient and washed three times with wash medium before experimentation [27]. Schistosomula were cultured at 37°C in modified Basch's medium (containing 10% Fetal Calf Serum) under 5% CO_2_ atmosphere [21],[28], either in the presence or absence of erythrocytes, for 3 h or 5 days. Parasites were washed in RPMI and stored in Trizol at −80°C until the total RNA was isolated following manufacturer's instructions. RNA quality, integrity checks and concentration were assessed using Nanodrop ND-1000 spectrophotometer and Agilent 2100 Bioanalyzer [29]. Due to the limited availability of schistosomula material, a single biological sample was used.

### Microarray construction

The design and construction of the schistosome microarray has been previously reported [30]. The microarray comprises of 19,222 target sequences printed twice from two independent probe designs, including 12,166 probes derived from *S. mansoni* contiguous sequences (contigs) and 7,056 probes derived from *S. japonicum* contigs. The putative genes designated from *S. mansoni* contiguous sequences were the primary but not exclusive focus of the analysis, and the genes derived from *S. japonicum* contigs were also fully considered. Further details of the microarray design and the normalised data from this study are presented in Tables S1 and S2.

### Microarray hybridisation and feature extraction

The methods used in microarray hybridisation and feature extraction have been previously reported [30] and followed the manufacturer's instructions (One-Color Microarray-Based Gene Expression Analysis Protocol; Version 5.5, February 2007 Agilent). For each sample 300 ng of total RNA was used to synthesise fluorophore-labelled cRNA using Cyanine 3-CTP (Agilent Technologies One Color Microarray Kit). Samples were purified using the Qiagen RNeasy kit. Cyanine-labeled cRNA samples were examined at A_260_ and A_550_ using a ND-1000 spectrophotometer to determine yield, concentration, amplification efficiency and abundance of cyanine fluorophore. CY3c (1.65 µg aliquot) was incubated with the fragmentation mix (Agilent Technologies) for 30 min at 60°C. Samples were then combined with 2× Gene Expression Hybridisation Buffer HI-RPM, mixed and applied to a gasket slide pre-positioned in a hybridisation chamber (Agilent Technologies), placed in a hybridisation oven and incubated for 17 h at 65°C. Each hybridisation was performed in duplicate as a technical replicate. After hybridisation, microarray slides were washed using the standard protocol (Agilent Technologies) and scanned on an Agilent microarray scanner at 550 nm. The “tag image format files” (tiff) produced by the scanner were loaded into the image analysis program Feature Extraction 9.5.3.1 (Agilent Technologies) to establish standardised data for statistical analysis. All microarray slides were checked for background evenness by viewing the tiff image on Feature Extraction.

### GeneSpring analysis

Feature extracted data were analysed using GENESPRING software, version 7.3.1 (Agilent Technologies/Silicon Genetics). Duplicated microarray data (technical replicates) were normalised using the GeneSpring normalisation scenario for “Agilent FE one-color” which included “Data Transformation: Set measurements less than 5.0 to 5.0”, “Per Chip: Normalise to 50th percentile” and “Per Gene: Normalise to median”. Samples were also normalised to individual samples depending on the comparison to be made.

To compare developmental effects of the transformation from cercariae to schistosomula, 3 h schistosomula with erythrocytes and 5 day schistosomula with erythrocytes were normalised to expression levels of cercariae. To determine the effects of culturing schistosomula with erythrocytes, 3 h schistosomula with erythrocytes were normalised to 3 h schistosomula without erythrocytes; similarly 5 day schistosomula with erythrocytes were normalised to 5 day schistosomula without erythrocytes. Data sets were further analysed using published procedures [30],[31] consisting of methods related to one-colour experiments and utilised the signal intensity (gProcessedSignal) values determined using Agilent's Feature Extraction software including aspects of signal/noise ratio, spot morphology and homogeneity. ProcessedSignal represents signal after localised background subtraction and includes corrections for surface trends. Features were deemed *Absent* when the processed signal intensity was less than two-fold the value of the processed signal error value. Features were deemed *Marginal* when the measured intensity was at a saturated value or if there was a substantial amount of variation in the signal intensity within the pixels of a particular feature. Features that were not *Absent* or *Marginal* were deemed *Present*. Data points were included only if *Present* and contigs were retained if all data points were *Present*.

### Secretory signal sequence analysis

Transcripts that were at least two-fold up-regulated in a particular experiment were searched against a database consisting of the full protein datasets from the *S. japonicum* [32] and *S. mansoni* [4] genome sequencing projects using BLASTX. Protein hits with an identity greater than 95% were used as the protein translation of the EST and analysed using local versions of SignalP 3.0 [33] for secretory signal sequences and TMpred (http://www.ch.embnet.org/software/TMPRED_form.html) for transmembrane domains. Transcripts that had no high scoring identities in the predicted protein database were searched against the NCBI non-redundant protein database using BLASTX. If the top scoring protein had a bit score greater than 50 the reading frame of the blast translation was used to translate the EST into protein sequence. The protein sequence was then analysed using SignalP and TMpred.

### Real time PCR

Gene expression patterns of a subset of genes were validated using real time PCR. Genes were chosen to span a range of biological functions and life cycle expression patterns, with a focus on including genes that encode known secreted/membrane proteins of interest as vaccines and drug targets, such as tetraspanins and intestinal proteases. Complementary DNA was synthesised from total RNA using a QuantiTect whole transcriptome kit (Qiagen). All cDNA samples were synthesised from aliquots of the same total RNA and used for the microarray hybridisations at a concentration of 50 ng/µl quantified using a Nanodrop ND-1000 spectrophotometer. Subsequently, 1 µl aliquots were combined with 10 µl of SYBR Green, 3 µl of water and 2 µl (5 pmol) of the forward and reverse primers in a 0.1 ml tube. All reactions were performed on a Rotor-Gene 3000 real time thermal cycler (Corbett) and analysed using Rotor Gene 6 Software (Corbett). In order to minimise indiscriminate binding of double-stranded DNA, which can produce readings in the “no template” controls, separate reverse transcription and PCR steps were included. Primer sets used are described in Table S3. The housekeeping gene TC15682 (DNA segregation ATPase) [34] was used for primary normalisation for all experiments. This housekeeping gene was selected from the initial microarray data since it was consistently unchanged throughout all of the comparisons made. Each experiment was performed in duplicate and the confidence threshold (CT) of the second set was normalised to the first set before evaluation. The analysis of correlation between microarray and quantitative PCR was performed in Graphpad Prism Version 5 (Graphpad Software Inc.) and was based on a previously published analysis [35]. To correlate results from microarray and quantitative PCR platforms, we first determined whether the data were distributed normally. This involved the use of both the “D'Agostino & Pearson omnibus normality test” and “Shapiro-Wilk normality test”. Both tests indicated that the data were not normally distributed; thus a Spearman correlation (Rho) was employed. All methods used an alpha value of 0.05.

### Accession numbers

Supplementary Information (RAW Data) has been submitted at GEO- Gene Expression Omnibus, (http://www.ncbi.nlm.nih.gov/geo/) with accession numbers for the platform GPL7160, and series GSE18335.

## Results

Two questions were addressed using the microarray analysis of *S. mansoni* schistosomula cultured for 3 h and 5 days.

1. Which genes undergo changes in expression during the transition from cercariae to 3 h schistosomula to 5 day schistosomula?
2. What effect does the inclusion of erythrocytes have on gene expression during culture?

### Genes modulated during the transformation of cercariae to schistosomula

**Figure 1:**
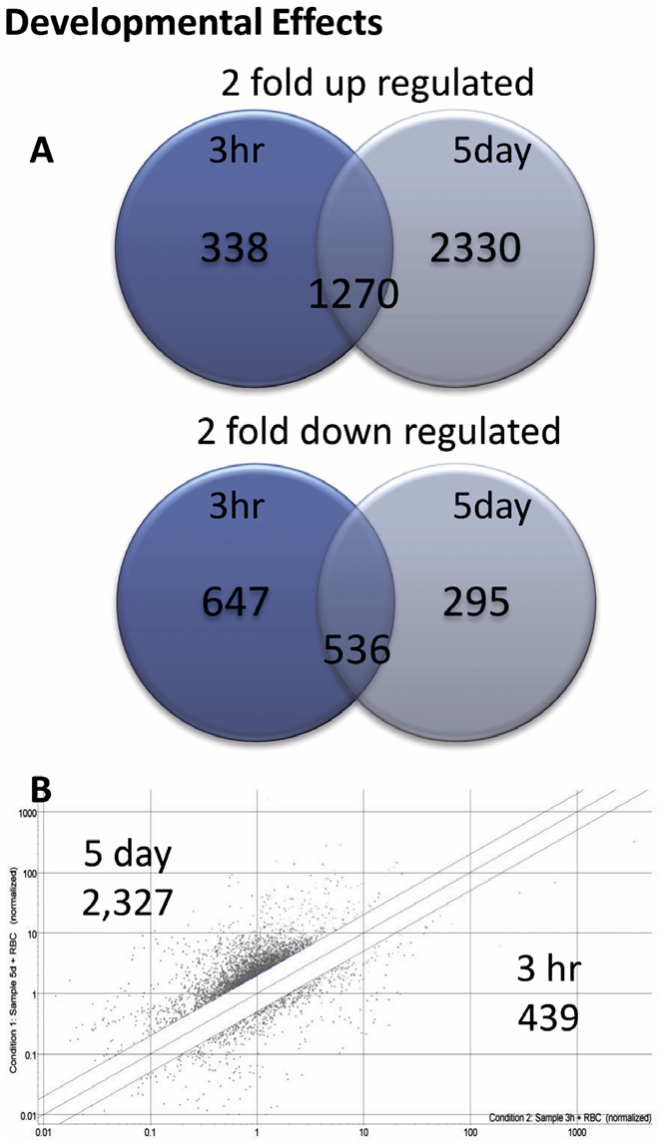
The number of genes differentially expressed between cercariae and 3 h or 5 day cultured *S. mansoni* schistosomula. (A) The number of genes either up- or down- regulated >2 fold in 3 h or 5 day schistosomula relative to gene expression in cercariae. Numbers in the overlapping region of the Venn diagram indicate genes that were differentially expressed at 3 h and 5 days. (B) Scatter plot of individual gene signal intensity of 3 h and 5 day schistosomula, with a 2-fold cut off, demonstrating the change in gene expression between the two time points.

Three hour and 5 day schistosomula were cultured in the presence of erythrocytes and the subsequent microarray data were normalised to the gene expression of cercariae. Data were filtered for each replicated data point for each of the 38,444 probes (19,222 contigs) on the microarray. Data points were filtered to preserve signals that were flagged during the feature extraction process as *Present* in all hybridisations; this resulted in the retention of 13,466 probes (7,764 contigs). A final cut-off was applied to the microarray data generating lists of genes that were ≥2-fold up- or down- regulated (relative to cercariae) for both 3 h and 5 day schistosomula. The number of genes (contigs) that were ≥2-fold up-regulated in the 3 h and/or 5 day schistosomula were 1,608 at 3 hours and 3,600 at 5 days, with 1,270 genes maintaining up-regulation at both time points (Figure 1A). Fewer genes were down-regulated ≥2-fold, with 1,183 at 3 hrs and 1,343 at 5 days; 831 genes remained down-regulated at both time points.

**Figure 2:**
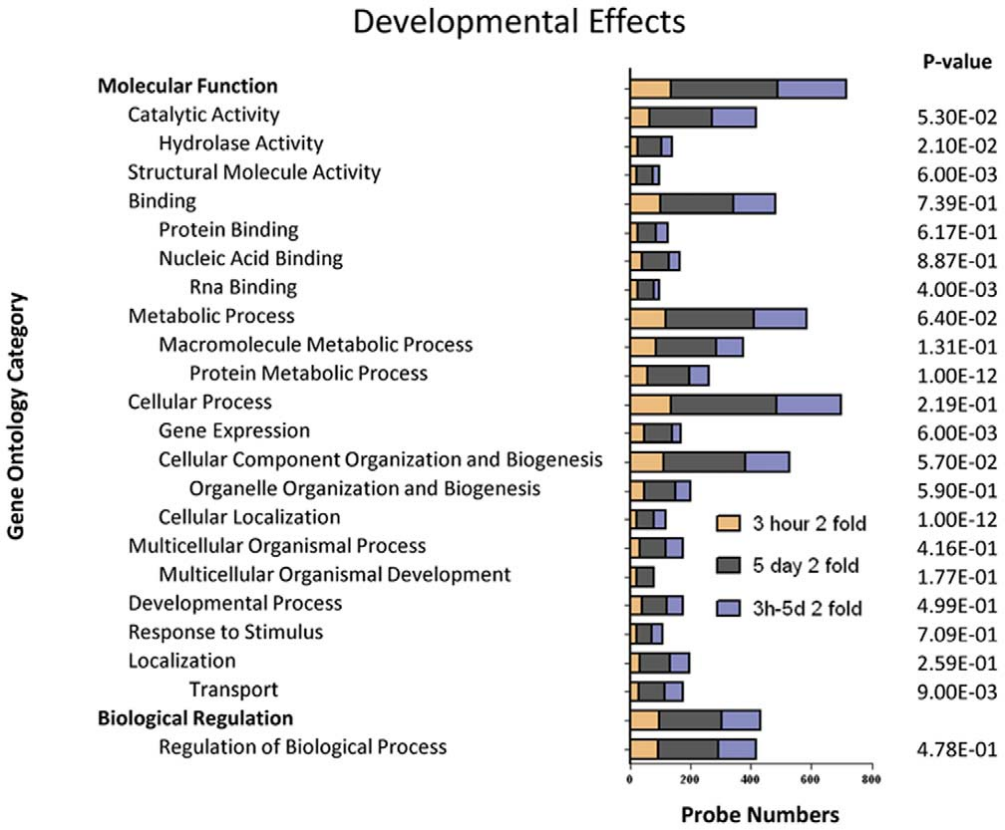
Major gene ontology categories up-regulated during schistosomula transformation. Gene ontologies of up-regulated genes during the transformation of *S. mansoni* cercariae to 3 h schistosomula, cercariae to 5 day schistosomula, and from 3 h to 5 day schistosomula. The P-value indicates the significance of the gene ontology category based on the number and degree of differentially expressed genes within each category. Categories with a P-value ≤0.05 were deemed significant; to show the position of these sub-categories within the GO tree hierarchy, parent categories with a P-value >0.05 were also included. Categories with P-value >0.05 may indicate that only a small percentage of the genes were differentially expressed.

The differential fold-change occurring between 3 h and 5 day cultured schistosomula was determined by plotting the signal intensity of the two time points and applying a >2 or <0.5 cut-off to represent a 2-fold up- or down- regulation. A larger number of genes were up-regulated during the transition from 3 h to 5 days (2,327 genes) compared with those that were down-regulated during this time period (439 genes) (Figure 1B). Examples of differentially expressed genes with novel annotations as well as the expression levels of genes encoding surface proteins of recently described vaccine antigens are presented in Table 1. Genes of particular interest that were highly up-regulated during the transition from cercariae to 3 h and 5 day schistosomula included the tegumental tetraspanin *Sm*-TSP-3 which was up-regulated almost 1,600-fold between cercariae and 5 day schistosomula. Other genes encoding tegument proteins that were upregulated included the Sm22.6 tegument associated antigen (137-fold upregulated at day 5) and a protein with homologues from the tegument of other flukes [36] and with low sequence identity to protein kinases (TC7982, 68-fold increased expression at day 5). Genes encoding two additional tegument proteins were rapidly up-regulated at 3h (annexin 7-fold and cytosol aminopeptidase 14-fold) and then further up-regulated by day 5 (annexin 21-fold and aminopeptidase 45-fold). A gene with sequence similarity to fasciclin 1 was up-regulated 100-fold in day 5 schistosomula (TC14173); the full-length protein (Smp_141680) was obtained from *S. mansoni* GeneDB (http://www.genedb.org/genedb/smansoni/) and contained two predicted transmembrane domains. Genes encoding intestinal proteases with known or suspected roles in digestion of the blood meal were highly upregulated by day 5 and included cathepsin B (65-fold), cathepsin L (37-fold), cathepsin D (13-fold) and cathepsin C (11-fold). The third most highly up-regulated gene in 5 day schistosomula was a S01 family serine protease (TC16843; 108–150-fold), although its anatomical location, and therefore potential function, has not been determined. Some of the genes that were up-regulated very quickly after transformation (by 3 h), included cathepsin B (10-fold), cathepsin L (2-fold) and cathepsin D (2-fold). At a more global level, the transformation between cercariae and 3 h/5 day schistosomula resulted in up-regulation of genes encoding a wide range of gene ontology (GO) categories including stress effectors (HSP70 and mitochondrial dicarboxylate transporter), enzymes involved in digestion of the blood meal (proteases) and iron storage (ferritin 2 (somal ferritin); upregulated 135-fold) (Figure 2). Other noteworthy genes included transmembrane transporters of zinc that were elevated in both 3 h and 5 day schistosomula (TC7518 ZNT4_RAT Zinc transporter 4, 3-fold at 3 h and 9-fold at 5 day; TC18440 solute carrier family 30 zinc transporter member 6, 2- fold at 3 h and 4-fold at 5 days).

**Table 1:** Examples of selected genes during the development of 3 h and 5 day schistosomula of *S. mansoni*.

			Developmental Effects Normalised to Cercariae		Culturing Conditions Normalised to no RBCs	
Category	Contig	Gene Description	3 h	5 day	3 h	5 day
***Tegumental***	TC18051	TSP-3	1.3	1598.2	1.1	1.8
	TC7927	SM22.6 antigen, calcium-binding protein	2.2	137.2	1.5	1.1
	TC7982	SJCHGC09029 tegumental protein	0.7	68.4	1.2	1.5
	TC13829	cytosol aminopeptidase, cb283 protein	13.9	44.9	1.6	1.5
	TC14162	Annexin	7.2	21.4	1.9	1.3
	TC13666	200 kDa Surface protein	1.1	8.1	1.3	1.6
	TC7639	TSP-1	1.6	3.7	1.6	1.4
	TC14339	Phosphodiesterase	2.0	2.7	1.6	1.6
	TC13787	Voltage dependent anion channel	1.3	2.5	1.5	1.4
	TC10775	Sm29	1.1	1.8	1.7	1.6
	TC13732	TSP-2	1.1	1.1	1.4	1.6
	TC7516	Sodium/potassium transporter (SNaK1)	0.2	0.9	1.2	1.7
	TC17273	Alkaline phosphatase II	0.1	0.1	1.2	1.5
	TC8556	TSP-4	0.1	0.1	1.2	2.1
	TC16328	Alkaline phosphatase III	0.0	0.1	1.8	2.0
***Gut Function***	TC13586	cathepsin b	9.5	65.3	1.2	1.2
	TC10560	cathepsin b	1.9	57.6	1.5	1.6
	TC16793	cathepsin l	2.1	36.9	F	F
	TC12166	cathepsin a	1.2	29.4	1.3	1.1
	TC12223	cathepsin l	2.1	26.9	1.8	1.6
	TC18677	cathepsin d	1.9	12.9	1.7	1.2
	Contig8499	cathepsin c	1.6	11.4	1.0	1.6
	TC7388	cathepsin d	1.6	5.4	1.4	1.7
	TC7801	cathepsin c	1.1	4.3	1.7	1.4
***Stress***	TC7378	elastase 2a	2.8	0.2	1.4	3.8
	TC16535	hsp70 homologue	4.7	3.3	1.2	3.4
	TC16538	heat shock 70kda protein 8	24.0	4.3	2.0	3.0
	TC11425	sulfite oxidase	0.4	1.2	1.3	2.8
	TC16843	elastase 2b	0.5	82.4	1.7	2.5
	TC11632	endonuclease-reverse transcriptase	0.3	0.9	0.5	2.2
	TC18034	mitochondrial dicarboxylate transporter	1.2	2.8	1.3	2.0
	TC10475	HSP60	6.9	14.1	0.8	1.7
	TC7721	oligosaccharyl transferase	1.4	2.1	1.4	1.7
	TC16965	Glucosidase II	1.7	3.5	1.1	1.7
	TC14312	minichromosome maintenance complex component 2	4.0	11.9	1.6	1.5
	TC11759	dehydrogenase reductase 2	0.6	1.5	1.2	1.3
***Development***	TC7600	Ferritin-2 heavy chain	4.1	134.6	1.7	19.8
	TC13678	low-density lipoprotein receptor (vitellogenin receptor)	1.8	13.0	0.3	6.2
	TC12477	serine protease	6.2	1.3	1.4	5.7
	TC6892	putative heme-binding protein	0.1	0.0	3.1	4.4
	TC10469	mKIAA1249, development and differentiation-enhancing	2.9	3.1	2.1	4.4
	TC19347	Regulator of presynaptic morphology protein 1	5.5	3.2	4.9	4.4
	TC11904	androgen receptor	1.5	3.1	1.5	4.4
	TC11453	cdc42 binding protein kinase alpha (dmpk-like)	0.9	2.1	1.8	4.4
	Contig5174	ftz-f1 interacting protein	3.3	7.2	1.8	4.3
	TC17814	spindle pole body component 97	16.5	2.9	1.5	4.3
	TC15784	glycogenin	0.6	0.5	1.6	3.5
	TC13278	bestrophin 2	1.0	2.1	1.4	3.4
	TC16714	epidermal growth factor receptor	1.4	2.7	0.5	2.5
	TC16824	Paramyosin. [Blood fluke], complete	1.2	4.0	1.5	1.4

Microarray data are presented as fold changes relative to the period of culture (cercariae for the developmental changes) or culture condition (presence of erythrocytes). Genes are presented under broad themes based on gene function. F = Gene failed filtering in that particular comparison.

### Changes in gene expression of schistosomula in the presence of erythrocytes

**Figure 3:**
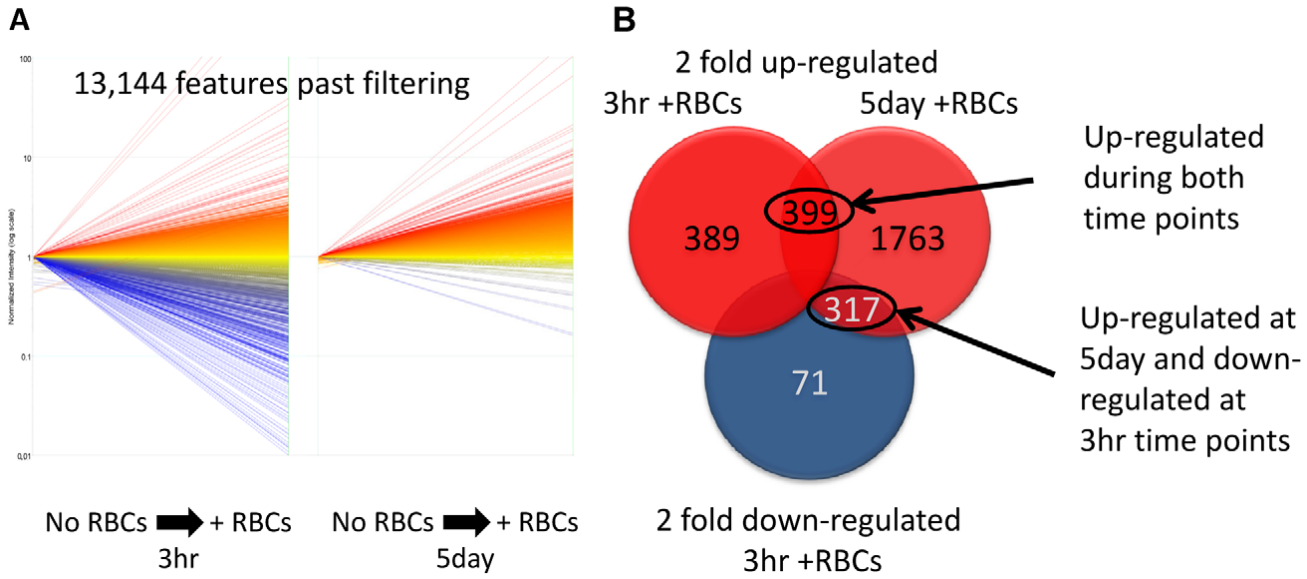
Number of genes differentially expressed in 3 h and 5 day cultured *S. mansoni* schistosomula in the presence of erythrocytes. (A) Scatter plot of the overall change in expression of 3 h and 5 day old schistosomula, cultured in the presence of erythrocytes and normalised to parasites cultured without erythrocytes. Relative up-regulation is shown in red, and relative down-regulation in blue. No differential gene expression is presented in yellow (B) Number of genes either up- or down-regulated >2-fold in 3 h or 5 day schistosomula cultured in the presence of erythrocytes relative to parasites cultured without erythrocytes; numbers in the overlapping region of the Venn diagram indicate genes that were differentially expressed in more than one group. Relative up-regulation is shown in red, and relative down-regulation in blue. Number of up-regulated genes in both time points (3 h and 5 day) and number of genes up-regulated at 5 day but down-regulated at 3 h time points are circled and annotated.

**Figure 4:**
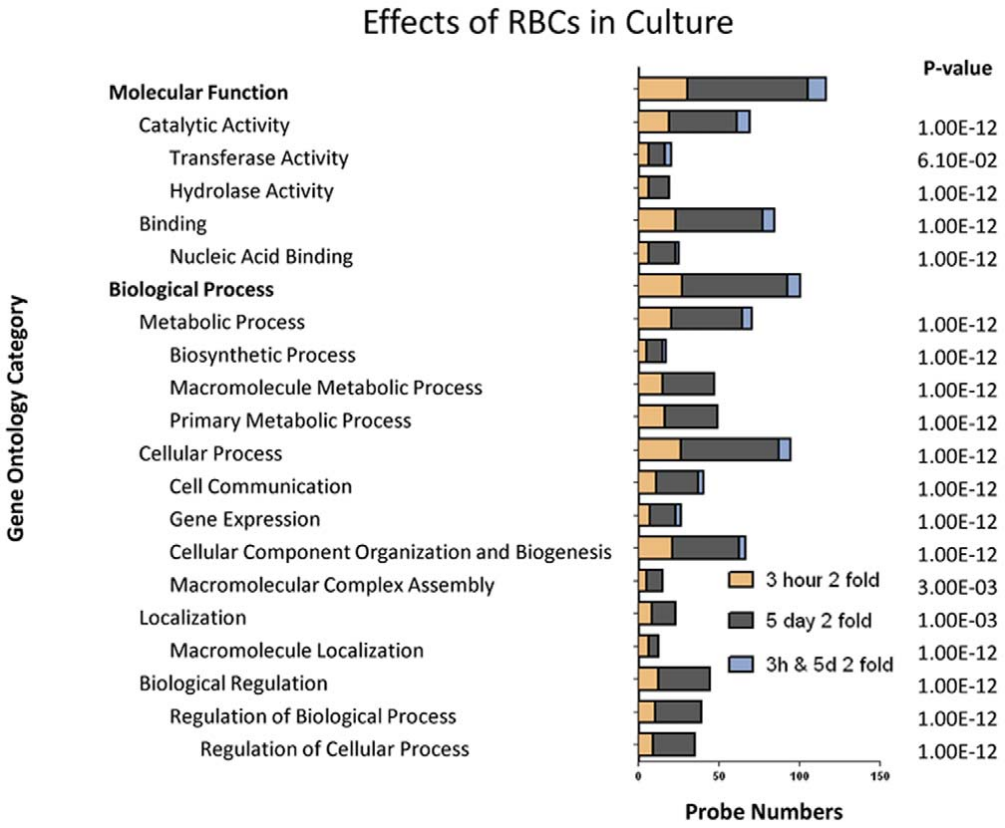
Major gene ontology categories of genes that were differentially expressed due to culture of *S. mansoni* schistosomula in the presence of erythrocytes. Gene ontologies representing the up-regulated genes in 3 h or 5 day schistosomula, and in both 3 h and 5 day schistosomula due to the presence of erythrocytes in the culture medium. P-values indicate the significance of the number and degree of differential expression within each category. Categories with a P-value ≤0.05 were deemed significant; to show the position of these sub-categories within the GO tree hierarchy, parent categories with a P-value >0.05 were also included. Categories with P-value >0.05 may indicate that only a small percentage of the genes were differentially expressed.

Schistosomula cultured for 3 h or 5 days were maintained in the presence or absence of erythrocytes and the subsequent microarray data were normalised to the gene expression of parasites cultured without erythrocytes. Data were filtered for each replicated data point for each of the 38,444 probes (19,222 contigs) on the microarray. Data points were filtered to preserve signals that were flagged during the feature extraction process as *Present* in all hybridisations; this resulted in the retention of 13,140 probes (7,599 contigs) (Figure 3A). A final cut-off was applied to the microarray data generating lists of genes that were ≥2-fold up- or down- regulated (relative to culture conditions without erythrocytes) for both 3 h and 5 day cultured schistosomula. A total of 788 genes at 3 h and 2,479 at 5 days were ≥2-fold up-regulated; 399 genes were up-regulated at both time points (Figure 3B). A small number of genes (388) were down-regulated in 3 h schistosomula. A small proportion of genes that were up-regulated at 5 days were down-regulated initially at 3 h (317 genes). Examples of novel genes and gene whose expression was up-regulated during the development of schistosomula from the cercarial stage are presented in Table 1, and further examples of novel up-regulated genes due to the presence of erythrocyte either at 3 h or 5 day schistosomula are presented in Table 2. The presence or absence of erythrocytes had little to no effect on the expression of genes encoding intestinal proteases, and of the genes encoding exposed tegument proteins, only TSP-4 and alkaline phosphatase III were up-regulated (2-fold) in the presence of erythrocytes. However, the inclusion of erythrocytes induced more obvious expression changes in genes involved in development and, to a lesser extent, the stress response (Table 1). These included genes encoding proteins involved in iron storage (ferritin-2, 20-fold increase), divalent metal transporter, heme-binding protein, proteolytic enzymes (serine protease), a fatty acid receptor, glycogen formation (glycogenin) and other components potentially involved in cellular differentiation/morphology. An overview of the differentially expressed genes in the presence/absence of erythrocytes is presented as GO categories in Figure 4.

**Table 2:** Examples of genes that were up-regulated either in schistosomula of *S. mansoni* cultured for 3 hours or 5 days in the presence of erythrocytes.

		Developmental Effects Normalised to Cercariae		Culturing Conditions Normalised to no RBCs	
Contig	Gene Description	3 h	5 day	3 h	5 day
TC13678	similar to low-density lipoprotein receptor [*Chiloscyllium plagiosum*], partial (3%)/SJCHGC03880 vitellogenin receptor	1.8	13.0	0.3	6.2
TC10050	SJCHGC04667 protein [*Schistosoma japonicum*], hypothetical protein	0.4	1.5	0.5	6.0
TC7156	extracellular serine-threonine rich protein	4.2	7.8	0.4	3.9
TC7102	mucin-associated surface protein (MASP), putative [*Trypanosoma cruzi*]	0.7	6.5	0.1	3.5
TC13651	similar to seven transmembrane helix receptor [*Homo sapiens*], partial (5%)	0.5	1.2	0.6	3.4
TC10651	SJCHGC03199 protein [*Schistosoma japonicum*], similar to lipopolysaccharide-induced TNF factor(*Gallus gallus*)	1.0	1.9	0.1	3.2
TC7868	similar to mucin 5 [*Homo sapiens*], partial (2%)	0.5	8.3	0.1	3.1
TC8184	weakly similar to Cullin homolog 3 (CUL-3). [Human], partial (30%)/SJCHGC07208	1.1	1.6	0.3	3.0
TC13461	homologue to PAXILLIN-LIKE PROTEIN [*Dictyostelium discoideum*], partial (4%)/mucin 17	2.3	6.5	0.1	2.9
TC16784	glucan 1 4-beta-glucosidase [*Xanthomonas axonopodis* pv. citri str. 306], partial (1%)/CG33300-PA [*Drosophila melanogaster*]	1.1	5.3	0.3	2.8
TC15511	activin receptor type	0.7	6.0	0.1	2.5
TC16880	similar to MAP kinase [*Strongylocentrotus purpuratus*], partial (85%)/mitogen-activated protein kinase 3	0.6	0.5	0.3	2.5
TC10495	homologue to cathepsin B1 isotype 1 [*Schistosoma mansoni*], partial (27%)	F	F	1.3	2.3
TC16719	epidermal growth factor receptor [*Schistosoma mansoni*], partial (4%)	1.3	4.8	0.2	2.1
TC8556	TSP-4	0.1	0.1	1.2	2.1
TC10971	weakly similar to unnamed protein product [*Homo sapiens*], partial (9%)/SJCHGC05056 epsin 2	1.4	2.3	1.3	2.0

Microarray data are presented as fold changes relative to schistosomula cultured for the same period in the absence of erythrocytes.

### Validation of mRNA expression using quantitative PCR

**Figure 5:**
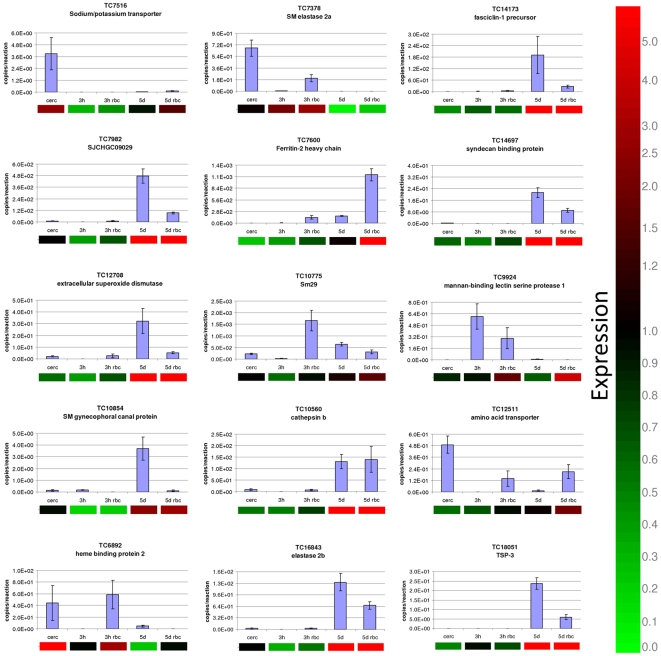
Validation of a subset of differentially expressed genes in 3 h and 5 day schistosomula of *S. mansoni* cultured in the presence or absence of erythrocytes. Quantitative real time PCR data, expressed as copy number per reaction, are presented as bar graphs, while the corresponding microarray data are shown below the graphs as heat maps. Microarray gene expression is indicated by up-regulation (red), down-regulation (green) or unchanged (black). The microarray data were not normalised to any sample.

To validate the microarray transcription data, mRNA expression profiles for 15 genes from different functional categories were assessed using quantitative real time PCR. Two independent experiments were carried out for this validation. The relative differential gene expression obtained by microarray analysis and by quantitative PCR was similar for the majority of data points for the 15 genes assessed (Figure 5). There was a significant correlation of 0.9208 between the two data sets (Spearman's Rho, *P*<0.0001, *n* = 34).

### Up-regulation of genes encoding for secreted proteins

**Figure 6:**
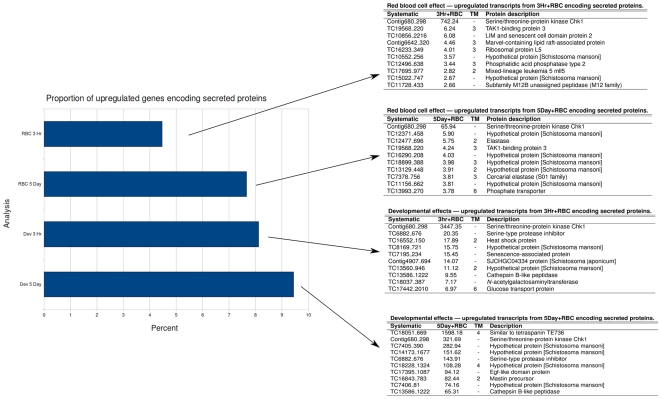
Percentage of genes encoding for secreted/membrane proteins that underwent ≥2-fold increased expression in each category. All genes that were up-regulated were screened for the presence of a signal peptide or anchor using SignalP; those ORFS with a signal peptide/anchor were then further screened for transmembrane (TM) domains using TMPred. The tables show the ten most highly upregulated genes in each category.

Genes that were upregulated ≥2-fold in any of the comparisons were assessed for the presence of signal peptides/anchors and transmembrane domains, suggestive of an extracellular location and therefore potentially involved in host-parasite interactions. Eight percent of genes that were up-regulated in 3 h old schistosomula (compared with cercariae) encoded for secreted proteins; all of these had 20-fold or less increases in expression except for contig 680_298, which was upregulated 3,447-fold in the absence of erythrocytes and an additional 742-fold in the presence of erythrocytes (Figure 6). Other genes up-regulated in 3 h parasites that encoded secreted proteins of interest included TC6882_676, a serine protease inhibitor that was up-regulated 2-fold and then an additional 144-fold in 5 day old parasites. More than 9% of genes that were up-regulated in 5 day parasites (compared to 3 hr parasites) encoded for secreted proteins; fold-changes in expression were generally higher than those seen in 3 h parasites, with the top ten ranging from 1,598-fold (TC18051_669 tetraspanin *Sm*-TSP-4) to 65-fold (TC13586_1222 cathepsin B) (Figure 6). Four of these top 10 most highly upregulated secreted proteins shared no identity with any proteins of known function. The complete list of genes encoding for secreted proteins that underwent ≥2-fold increased expression in any of the experimental groups is provided in Table S4.

## Discussion

In this study we have presented a comprehensive analysis of the transcriptional changes that are associated with two distinct and critical phases of the early maturation of the intra-mammalian stages of *S. mansoni*. We investigated the developmental changes that occur *in vitro* (1) in the first few hours after transformation as the cercarial glycocalyx is shed and the parasite adapts to an intra-mammalian environment, and (2) as schistosomula mature *in vitro* over a 5 day period. This developmental window corresponds to the *in vivo* phase of parasite migration from the skin (3 h) into the vasculature en route to the lungs (5 days). *In vitro* culture exposes the parasite to host serum (and erythrocytes) but does not entirely mimic intra-mammalian development due to the absence of tissue barriers to penetrate, a vascular system to navigate and a complete immune system to avoid. We also examined the effects on gene transcription when erythrocytes were present in the culture medium. For both analyses, we placed emphasis on genes that encode proteins at the host-parasite interface, namely exposed tegument proteins and key proteins involved in nutrient acquisition.

The development of newly transformed *S. mansoni* schistosomula over the first 5–7 days as they enter the vasculature and progress to the lungs represents what many believe to be a critical window of opportunity for vaccine-mediated protection [3],[37],[38]. At this stage the parasite presents a distinct suite of proteins on its tegument and has not yet become fully cloaked in host-derived molecules [39]. Moreover, juvenile schistosomula are more susceptible to antibody-dependent cellular cytotoxicity [40] than are older schistosomula and adult worms. We therefore reasoned that genes that are highly expressed at this stage, particularly those encoding secreted and membrane proteins, are worthy targets for the development of vaccines and new drugs. Recent microarray based studies of *S. mansoni* [41] and *S. japonicum* [30] have profiled the gene expression patterns of a wide range of lifecycle stages. However, one aspect of the parasite lifecycle that has not been examined in detail for either species is the transformation of the cercarial stage to the “skin” schistosomula, and the maturation of these parasites into “lung” schistosomula. Dillon and co-workers [25] compared cDNAs from 2 day and 7 day old cultured schistosomula with a control pool of mixed lifecycle stage cDNAs. They utilised a cDNA microarray consisting of 3,088 unique contigs and identified broad categories of differentially expressed genes in the schistosomulum stage, including energy metabolism, cytoskeletal organisation, protease activity and chromosome remodelling. Our study differs from that of Dillon's in a number of ways; (1) we focused on different time points of cercarial transformation and maturation, 3 hours and 5 days post-transformation, since these times better approximate the skin and lung schistosomula stages of *S. mansoni*; (2) we compared the gene expression profiles of each developmental stage to the stage preceding it, i.e. schistosomula to cercariae instead of using a pooled multi-stage cDNA preparation for determining baseline expression; and (3) we utilized an array consisting of 12,166 *S. mansoni* unique contigs.

Our results indicate that the transcriptional changes that accompany the development of cercariae to 3 hour and then to 5 day schistosomula are mostly found in the up-regulation of genes associated with many different molecular functions, but particularly catalytic activity and binding. Considerably fewer genes were down-regulated in the first 3 hours after transformation, most likely representing genes that are important for the free-swimming cercariae and are no longer required by the intra-mammalian stages. Jolly and co-workers found that the most highly up-regulated genes in cercariae (compared to other life cycle stages) were mitochondrial in nature representing the increased energy requirements needed for motility during this stage [41]. This observation was paralleled in our study where a number of mitochondrial genes such as NADH subunit 2 and NADH subunit 5 (see Table S2) were down regulated (up to 25-fold) at 3 h post-transformation compared with the cercarial stage. We observed that most probes to actin or actin related genes and fibrillin 2 were upregulated in the schistosomula relative to cercariae. The differential expression of structural genes such as actin may reflect the increased levels of tegument matrix generation and turnover as well as smooth muscle formation in newly transformed and growing parasites [42],[43].

We were particularly interested in the expression of genes encoding exposed proteins on the tegument of the parasite. Tetraspanins are four transmembrane spanning proteins represented by at least 5 distinct members in the tegument membranes of the adult parasite [2],[5],[6],[44]. *Sm*-TSP-1 and TSP-2 are efficacious vaccines [24]; *tsp-1* mRNA was up-regulated almost 4-fold in 5 day schistosomula but *tsp-2* expression levels did not change. However, one of the most highly up-regulated gene on the entire microarray (TC18051), undergoing 1,598-fold up-regulation between 3 h and 5 day cultured parasites, was another tegument tetraspanin [6] that we have termed *Sm-tsp-3* [2]. This considerable up-regulation of *tsp-3* was confirmed by quantitative PCR. Genes encoding other tegument proteins that were significantly up-regulated during this developmental period included Sm22.6, an unknown protein, and annexin. Sm22.6 is an inhibitor of human thrombin [45], and its up-regulation upon entry into the host vasculature likely represents an important survival strategy. Recombinant Sm22.6 confers partial protection as a vaccine in murine studies [46] but it is a major target of IgE in infected people [47], so its utility as a vaccine is likely limited. The third most highly up-regulated gene in 5 day schistosomula was a S01 family serine protease (150-fold), sharing identity with cercarial elastases that digest connective tissue proteins [48],[49]. Unlike the cercarial elastases where mRNA expression is highest in sporocysts and is switched off in cercariae and intra-mammalian stages [50], this new serine protease likely plays a distinct role due to its up-regulation in maturing schistosomula. We also detected 100-fold up-regulation in 5 day schistosomula of a gene encoding for a homologue of fasciclin 1, a family of GPI- anchored proteins that mediate cell adhesion through an interaction with alpha3/beta1 integrin [51].

As schistosomula mature their gastrodermis forms and they begin to ingest blood as a source of nutrition. The digestive process begins with haemolysis where erythrocytes are ingested and lysed by the action of a haemolysin(s) within the oesophagus and intestine [52]. Saposin-like proteins (SAPLIPs) are candidate pore-forming haemolysins in the schistosome gut [53], and we identified a SAPLIP that was highly up-regulated in 5 day schistosomula (TC_14899, 25-fold up-regulated). Two other SAPLIP-encoding genes, TC10647 and TC10646, were also up-regulated (5- and 3-fold respectively) in 5 day schistosomula, suggesting a role for this protein in blood feeding, either via haemolysis or transport of lipids. An anion sugar transporter, distinct from the well characterised glucose transporter family from the *S. mansoni* tegument [54], was up-regulated 25-fold in 5 day schistosomula, although its site of expression (gut, tegument or elsewhere) has not yet been determined. Other intestinal genes that were up-regulated between 3 h and 5 day schistosomula were the digestive proteases. The role of cysteine and aspartic proteases in digestion of haemoglobin and serum proteins for nutritional support is well recognised [55],[56], and the increase in transcription of these genes as schistosomula begin to feed on blood supports their roles in this process.

Erythrocytes provide the parasite with an important nutritional source. However, other host factors also have an impact on parasite development; for example, the presence of insulin in the culture medium induced the increased expression in *S. japonicum* of many genes related to sexual reproduction and protein translation in general [57]. In this current study, serum (which contains insulin) was present throughout during culture, but other components, such as glycoproteins or glycolipids, derived directly from the erythrocytes may have impacted on gene expression. The up-regulation of a low-density lipoprotein receptor in the presence of erythrocytes may be influential in the development of the female reproductive system since this protein is also a putative vitellogenin receptor (Table 1). Lipids are a component of the female reproductive tract in schistosomes, as indicated previously by the localisation of a fatty acid-binding protein within lipid droplets of vitelline cells [58]. The most commonly encountered transcripts that were up-regulated in the presence of erythrocytes were of retroviral/retrotransposon origin. This is not surprising, as we have previously reported the up-regulation of retrotransposons in geographical isolates of schistosomes [59], and their up-regulation here in the presence of erythrocytes may be in response to environmental changes/stress.

Other genes that were up-regulated in 5 day schistosomula in the presence of erythrocytes included at least three distinct mucins that were >3-fold up-regulated, and an activin receptor, which have been described from secretions of schistosome eggs and cercariae [60] and the tegument [61]. Glycosidases were also upregulated, an example being glucan 1 4-beta-glucosidase (TC16784). This was 2.8 fold up-regulated in 5 day schistosomula in the presence of erythrocytes compared to their erythrocyte-free cultured counterparts, further implying a general increase in expression of genes involved in acquisition and metabolism of erythrocyte proteins, lipids and glycans. Only one of the digestive proteases showed greater than 2-fold increased expression in the presence of erythrocytes (TC10495, homologue to cathepsin B1), implying that their up-regulated expression is predominantly independent of erythrocytes (and therefore haemoglobin), but instead is dependent on serum proteins. Selected examples of the most highly up-regulated (non-retroviral) genes in the presence of erythrocytes in 5 day schistosomula are provided in Table 2.

The transformation from cercaria to schistosomulum requires the adaption of the parasite to radically different environments and subsequent large scale cellular differentiation and growth. This stressful process is reflected by the up-regulation of multiple stress related genes [62], including numerous heat shock proteins and minichromosomal maintenance complex component 2, all of which were up-regulated at 3 h and were maintained at elevated expression levels through day 5, relative to cercariae.

Another major feature of the maturing schistosomula is the extensive musculature that begins to form beneath the tegument [42]. This increase in muscle tissue was reflected in our study by increased expression levels of a number of muscle related genes including paramyosin, myosin light chain kinase and myosin light polypeptide 5 regulatory sequence (See Table S2). Paramyosin is of particular importance to intra-mammalian stages due to its dual function as a structural element of smooth muscle and its immunomodulatory function in schistosomula [63],[64],[65].

The tegument of *S. mansoni* schistosomula is the major target of the immune response of a population of putatively resistant (PR) individuals in *S. mansoni* endemic areas of Brazil [66], and in schistosomiasis resistant rats [67]. By identifying genes that are highly upregulated during this developmental process, particularly those that are accessible to antibodies such as the exposed tegument and gut proteins, a new suite of vaccine antigens can be produced and screened with sera from resistant hosts. We envisage this study as an initial screen for potential vaccine antigens and drug targets, to be followed by murine protection studies and functional analysis where approaches such as RNA interference could be used to verify the consequences of silencing these genes under different *in vitro* and *in vivo* conditions.

## Supporting Information

Table S1 Complete lists of contiguous sequences listed in the custom designed schistosome microarray manufactured by Agilent Technologies used in this study. Column A (ProbeID): Unique identifier of probe on the microarray. Column B (Sequence): Nucleotide sequence of the 60mer probe. Column C (EST Sequence): Complete nucleotide sequence of the assembled EST contig. Column D (TargetID): Contig designation for either *S. japonicum* (Contig) or *S. mansoni* (TC). Column E (Accessions): Genbank accession number corresponding to the EST sequences. Column F (Description-Nucleotide): BLASTn annotation result based on nucleotide sequence. Column G (GeneSymbols): Designation of primary or secondary probe design to the corresponding contig. Column H (Protein Homology): BLASTX annotation result based on protein sequence. Column G (Gene Ontology): Gene Ontology number and description.
(8.96 MB ZIP)

Table S2 Complete list of differentially expressed genes, shown on separate sheets of a Microsoft Excel File.
(7.41 MB XLS)

Table S3 Primer sets for real time PCR validation of a subset of genes that were differentially expressed in schistosomula under different culture conditions and/or relative to the cercarial stage.
(0.03 MB XLS)

Table S4 Genes encoding for secreted/membrane proteins that underwent ≥2-fold increased expression in each category. All genes that were up-regulated were screened for the presence of a signal peptide or anchor using SignalP; those ORFS with a signal peptide/anchor were then further screened for transmembrane (TM) domains using TMPred.
(0.15 MB XLS)
